# Interrelationships and cross‐pathways between αSyn and pTau aggregates. Vitro and vivo evidences

**DOI:** 10.1002/alz70855_103110

**Published:** 2025-12-24

**Authors:** Noelle Callizot, Laura Rouvière, Catherine Botto, Alexandre Wojcinski, Delphine Hardy, Alexandre Henriques

**Affiliations:** ^1^ Neuro‐Sys, Gardanne, France

## Abstract

**Background:**

The co‐occurrence of multiple protein aggregates, or even hybrid aggregates, is very often observed in many neurodegenerative diseases, corroborating their molecular cross‐links. Despite the emergence of studies indicating crossovers between Amyloid β (Aβ), hyperphosphorylated Tau protein (pTau) and alpha‐synuclein (αSyn), a thorough understanding of their mechanistic coupling and subsequent pathogenicity is still lacking.

It is well known that there are molecular interactions between these different proteins, and in particular between αSyn and pTau and Aβ. However, there is little evidence of a direct link between the formation of one and the appearance of the other. The close links between these proteins on their appearance was studied.

**Method:**

Primary cultures of hippocampal and dopaminergic neurons both were injured with Aβ oligomers or αSyn PFF. Mitochondrial and lysosomal stress were carefully studied. At different timing accumulation of toxic protein was observed (by ICC and protein analysis).

In vivo, aged mice (18 month old) were injured with Aβ or αSyn PFF by stereotaxic injection. Up to 6 weeks after the lesion, appearance of toxic proteins was following in different brain areas (SNpc, hippocampal area, amygdala, striatum).

**Result:**

We observed a strong correlation between toxic αSyn aggregates and pTau accumulation. Similarly, aggregated αSyn was found after Aβ injections into the hippocampus. Appearance of these various toxic aggregates was always preceded by strong oxidative stress and by lysosome defects.

**Conclusion:**

These results demonstrate the complexity of the relationships between these different proteins. We demonstrated that not only physical interaction are existing, but that each induced formation of the others leading to neuronal death.

